# Multisensory Enhancement of Cognitive Control over Working Memory Capture of Attention in Children with ADHD

**DOI:** 10.3390/brainsci13010066

**Published:** 2022-12-29

**Authors:** Biye Cai, Shizhong Cai, Hua He, Lu He, Yan Chen, Aijun Wang

**Affiliations:** 1 Department of Psychology, Research Center for Psychology and Behavioral Sciences, Soochow University, Suzhou 215123, China; 2 Department of Child and Adolescent Healthcare, Children’s Hospital of Soochow University, Suzhou 215025, China

**Keywords:** ADHD, cognitive control, working memory, attentional capture, multisensory processing

## Abstract

Attention deficit hyperactivity disorder (ADHD) is a common neurodevelopmental disorder in school-age children. Although it has been well documented that children with ADHD are associated with impairment of executive functions including working memory (WM) and inhibitory control, there is not yet a consensus as to the relationship between ADHD and memory-driven attentional capture (i.e., representations in WM bias attention toward the WM-matched distractors). The present study herein examined whether children with ADHD have sufficient cognitive control to modulate memory-driven attentional capture. 73 school-age children (36 with ADHD and 37 matched typically developing (TD) children) were instructed to perform a visual search task while actively maintaining an item in WM. In such a paradigm, the modality and the validity of the memory sample were manipulated. The results showed that under the visual WM encoding condition, no memory-driven attentional capture was observed in TD children, but significant capture was found in children with ADHD. In addition, under the audiovisual WM encoding condition, memory-matched distractors did not capture the attention of both groups. The results indicate a deficit of cognitive control over memory-driven attentional capture in children with ADHD, which can be improved by multisensory WM encoding. These findings enrich the relationship between ADHD and cognitive control and provide new insight into the influence of cross-modal processing on attentional guidance.

## 1. Introduction

Attention deficit hyperactivity disorder (ADHD) is a common childhood mental health disorder that is characterized by a pattern of inappropriate levels of inattentiveness, hyperactivity, and impulsivity [1]. The dysfunctional behavioral inhibition hypothesis proposes that the behavioral symptoms of ADHD are due to deficits in executive function [2]. Previous studies have demonstrated that children with ADHD exhibit impairment and poor performance in various executive function tasks [3,4,5]. For example, using an antisaccade task, Fernandez-Ruiz et al. (2020) found that children with ADHD showed longer reaction times and more antisaccade direction errors, indicating that they processed distracting information less efficiently and had impaired top-down inhibitory control [6].

Apart from inhibitory control, working memory (WM), as another essential component of executive function, has been found to be impaired in individuals with ADHD in many studies [7,8]. A meta-analysis found that children with ADHD exhibited deficits in both visuospatial and phonological WM tasks [9]. Neuroimaging studies have shown that patients with ADHD show abnormal activation in brain areas serving WM, such as the bilateral dorsolateral prefrontal cortex and the left frontoparietal area [8,10]. It must be noted that some evidence has revealed that WM impairments are closely associated with deficits in attentional control in patients with ADHD [11,12]. Using a visuospatial WM task, Luo et al. (2019) found a reduced N2pc during the encoding phase, a reliable index for attentional selection. The reduction in N2pc was related to the amplitude of the contralateral delayed activity (CDA), a component associated with the maintenance of WM [11]. However, the issue of whether children with ADHD exhibit abnormalities in the interaction between WM and attention remains poorly understood.

The biased competition model suggests that there is a close relationship between WM and selective attention, with a stimulus that matches internal representations stored in WM prioritized for attentional selection as neural representations encoding memorized features are already preactivated [13]. In line with this theory, numerous studies have adopted a dual-task paradigm, pairing a visual search task with a change detection task, and verified that stimulus-matching WM contents automatically capture attention, which is termed memory-driven attentional capture [14,15,16]. Nonetheless, another part of the study found no obligatory capture [17,18], and further studies found better performance under distractor-matched conditions when participants knew that the target never matches the features of previously memorized items [19,20]. These findings indicated that the deployment of attention to memory-matched stimuli could be strategically controlled to optimize visual search [21,22].

Meanwhile, utilizing WM representations to guide attention away from memory-matched distractors requires sufficient cognitive control [21]. The more attentional resources available for cognitive control, the weaker the attentional capture driven by memory-matched distractors [23,24]. This capture effect can even be reversed into a suppression effect when the top-down cognitive control signal is stronger than the priority signal [25,26]. For example, a recent study found that WM capacity was related to cognitive control ability. Individuals with high WM capacity were able to actively suppress memory-matched distractors, whereas individuals with low WM capacity were vulnerable to these distractors [27].

Previous studies have demonstrated that children with ADHD have weakened cognitive control and difficulty in filtering out interference by task-irrelevant information and they exhibit inefficient neurocognitive mechanisms during active suppression [6,28,29]. For example, Wang et al. (2016) found that children with ADHD have a reduced ability to suppress distractors, demonstrated by a reduced amplitude of the Pd component, which is related to inattentiveness symptoms [29]. Studies on spectrum analysis found that children with ADHD showed smaller midfrontal theta event-related synchronization compared to controls in a visual search task, indicating insufficient cognitive control in those with ADHD [30,31]. Additionally, recent fMRI studies have indicated that ADHD has reduced neural activity in a wide range of brain regions involved in cognitive control, including the temporal, parietal, and cingulate cortical regions [32,33,34]. All of these studies imply that children with ADHD may be susceptible to memory-matched distractors and show robust memory-driven attentional capture due to a less efficient cognitive control mechanism.

Taken together, the present study investigated whether children with ADHD have sufficient cognitive control to modulate memory-driven attentional capture. A dual-task paradigm combining a visual search task and a change detection task was adopted. Participants were asked to perform a visual search task while maintaining the identity of a memory item that could either appear as a distractor or not in the search display. Critically, the task was designed to provide strong motivation to exert cognitive control. First, between the memory display and the search display, a cue display appeared that provided prior knowledge of both the identity and the location of subsequent search items to maximize the motivation to guide attention away from a memory-matched distractor. Second, we did not include valid search trials (i.e., trials on which a memory sample appears as a target in the search display) to eliminate the motivation to attend to a memory-matched item.

In addition, it has been demonstrated that semantically congruent bimodal presentation can enhance cognitive control and facilitate subsequent cognitive processing [35,36]. More importantly, previous studies have shown that individuals with ADHD have early multisensory integration, and the multisensory benefits in individuals with ADHD are more prominent than those in controls during a discrimination task with semantically congruent multisensory stimuli [37,38]. Thus, the modality of the memory sample was manipulated in the experimental design. Based on these manipulations, we predicted a robust attentional capture effect (slower search in invalid trials than in neutral trials) in children with ADHD under the unimodal (visual) encoding condition due to insufficient cognitive control. Given the evidence that audiovisual stimulation can affect cognitive control, we predicted that the capture effect would decrease or disappear under the multimodal (audiovisual) encoding condition.

## 2. Methods

### 2.1. Participants

G*Power 3.1.9.7 [39] was used to estimate the sample size for a 2 × 2 × 2 three-way repeated measures analysis of variance (ANOVA) (estimated effect size f = 0.25, alpha = 0.05, power = 0.9), yielding that a sample size of *N* = 23 for each group was sufficient to achieve the desired effect size. A total of 73 children aged 7 to 12 years were enrolled in the study, including 36 children with ADHD and 37 age- and sex-matched TD children. Children with ADHD were recruited from the Children’s Hospital of Soochow University, and controls were enrolled from local primary schools. The diagnosis of ADHD was based on being clinically interviewed with the Diagnostic and Statistical Manual of Mental Disorders, 5th ed. (DSM-5) criteria by qualified psychiatrists. In addition, all children with ADHD met diagnostic criteria on the Swanson, Nolan, and Pelham-IV Rating Scale (SNAP-IV) completed by their parents [40]. The SNAP-IV has been reported to demonstrate good test-retest reliability and validity based on previous studies [41,42]. There were 3 children with ADHD comorbid with oppositional defiant disorder (ODD).

All children with ADHD met the following criteria: (a) ADHD diagnosis based on DSM-5 by qualified psychiatrists and moderate to severe scores on the SNAP-IV; (b) right-handed; (c) normal hearing and normal or corrected-to-normal visual acuity; (d) normal intelligence quotient (IQ > 25%) as estimated by the Raven Standard Reasoning Test (RSRT); (e) drug-naïve; and (f) no history of other neurological or psychiatric disorders. The inclusion criteria for the TD group were the same as for the ADHD group, with the exception of the ADHD diagnosis. None of the participants in the TD group had academic or behavioral problems based on parent and teacher reports. Of the 73 potential participants, 3 participants (1 ADHD child with ADHD and 2 TD children) were excluded due to poor performance (the z score of accuracy or RT was larger than 3). Therefore, 35 children with ADHD (mean age: 9.06 ± 1.35 years; 29 males and 6 females) and 35 TD children (mean age: 8.91 ± 1.27 years; 27 males and 8 females) were ultimately included. No significant group differences in age (*t*_68_ = 0.46, *p* = 0.65) and sex ratios (χ^2^_1_ = 0.36, *p* = 0.55) were observed between groups.

In accordance with the Declaration of Helsinki, all parents of children provided informed consent. The study was approved by the Academic Committee of the Department of Psychology, Soochow University, Suzhou, China.

### 2.2. Stimuli and Apparatus

The visual stimuli were obtained from a standard set of outlined drawing pictures [43]. The selected pictures consisted of 5 black-and-white line drawings of animals with 4.8° visual angle. The auditory stimuli consisted of verbalizations that corresponded to the visual stimuli (e.g., the sound of a dog barking was paired with a picture of a dog). All of the sound files, downloaded from a website (http://www/findsounds.com, accessed on 24 December 2022), were delivered binaurally via stereo headphones (model: ATH-WS990BT) and were standardized in amplitude.

The experiment was programmed and executed using E-prime 3.0 software (Psychology Software Tools, Inc., Pittsburgh, PA, USA) and presented on a 32-inch LCD monitor (120 HZ, 2560 × 1440 screen resolution) with a black background (RGB: 0, 0, 0). Participants sat at a viewing distance of approximately 57 cm and provided their responses using two two-key customized keyboards.

### 2.3. Design and Procedure

**Figure 1:**
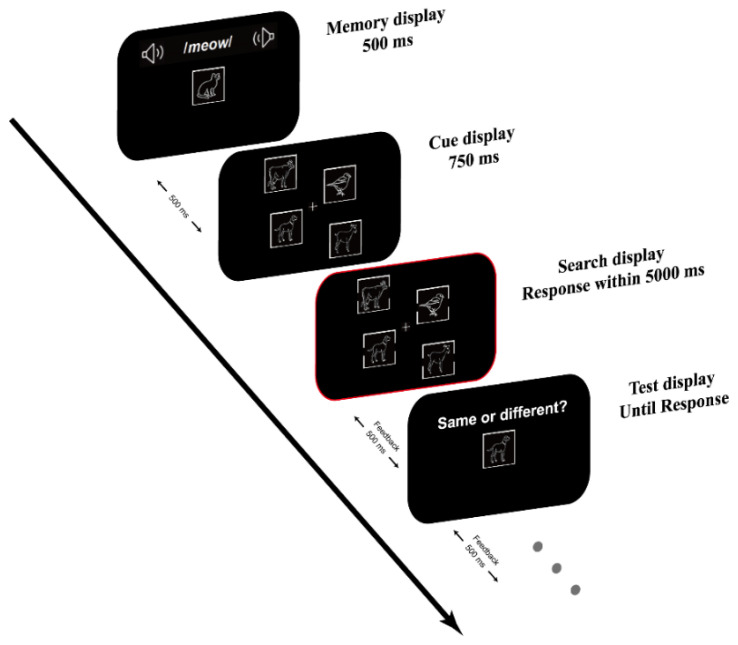
Procedure of events in a sample trial. Participants were asked to remember the memory sample (visual or congruent audiovisual) and complete a change-detection task in the test (here, the identity changed). In the retention interval, they searched and specified which side of the target had a larger gap. One of the distractors could match the identity or neither feature with the memory item. Between the memory display and the search display, a cue array was presented, providing prior knowledge of the identity and location of search items.

An example trial sequence is illustrated in Figure 1, our paradigm combined a visual search task with a change detection task. The change detection task included a memory sample and a memory test. There were two types of memory samples corresponding to the memory modality as follows: under the visual (V) condition, a line drawing of animals was presented inside a square box with a black frame located in the center of the screen; and under the audiovisual (AV) condition, a visual stimulus was presented simultaneously with a semantically related sound. During the memory test, a visual item appeared at the center of the screen as a memory test probe, either a match (50%) or non-match (50%) to the memory sample.

The cue array consisted of four visual items presented at either 1, 4, 7, 10 or 2, 5, 8, 11 o’clock locations around an imaginary circle (with a radius of 13°), with no items overlapping. The cue array was presented between the memory display and the search display, providing prior knowledge of the identity and location of search items. The setting of the cue array is useful to facilitate cognitive control to avoid distractions from memory-matched distractors [20,44].

The visual search items were identical to the cue items except that they had gaps on the left and the right side of the square box. The distractor had an equal-size gap (0.3° × 1.1°), whereas the target had a slightly larger gap (0.3° × 1.6°) on either the left or the right side. Half of the search trials were invalid trials, in which the memory sample reappeared as one of the distractors in the search display. The remaining half were neutral trials, in which memory items did not reappear in the visual search task.

Each trial began with a white fixation cross (1° × 1°) shown in the center of the center, after which the memory display was presented for a duration of 500 ms. Participants were required to memorize the memory item for a later memory test. After a blank interval of 500 ms, the cue display appeared for 750 ms. Following the cue display presentation, the search display appeared. Participants were required to judge which side of the target had a larger gap by pressing “F” for the left (50% of the total trials) and “J” for the right (50% of the total trials) with their left hands. Responses occurring outside the 5000 ms time interval following the search display onset were coded as incorrect. The memory test then appeared until the response. Participants needed to judge whether the probe stimulus was the same as the memory stimulus presented during the WM encoding stage with a key response (“yes” and “no” responses corresponded to the “Z” and “M” keys on the keypad, respectively). Participants received immediate feedback on accuracy. Each trial ended with a blank screen for a random duration ranging from 800 to 1000 ms. Participants were instructed to execute the search task as quickly and accurately as possible. The memory task had to be completed as accurately as possible, without time pressure.

Taken together, the present experiment was a 2 (memory modality: V vs. AV) × 2 (validity: invalid vs. neutral) × 2 (group: children with ADHD vs. TD children) three-factorial mixed design. Before the formal experiment, each participant was required to complete a practice block (16 trials) to get familiar with the task. The total experiment consisted of 128 trials, which were presented in blocks of 32 trials each (8 trials per condition). The validity was mixed randomly within the block, whereas the memory modality varied between blocks. The participants completed two V and two AV blocks, whose order was chosen randomly with equal probability from ABBA and BAAB design. The entire experiment lasted for about 30 min. Participants were informed in advance that memory-matched stimulus would never be a target, and the cue stimuli had the same identity and location as the subsequent search items.

### 2.4. Data Analysis

Statistical analyses were performed using JASP (Version 16). A two-tailed *p* < 0.05 was considered significant for all statistical analyses. The participants’ response time (RT) and accuracy were recorded and analyzed. Before analyzing the RTs for the search task, trials with incorrect responses in either the search or the memory task were excluded, as were trials with RTs ± 3 SDs beyond the mean RT within each condition for each participant. To explore whether there was a difference between children with ADHD and TD children, we conducted a 2 (memory modality: V vs. AV) × 2 (validity: invalid vs. neutral) × 2 (group: ADHD vs. TD) repeated-measures ANOVA on memory accuracy, search accuracy, and mean search RTs, respectively. After that, post hoc *t* tests based on Bonferroni correction were performed. Additionally, we conducted a 2 (memory modality: V vs. AV) × 2 (group: children with ADHD vs. TD children) repeated-measures ANOVAs on memory-driven attentional capture effect to determine how the capture effect (e.g., the RTs of invalid trials minus the RTs of neutral trials differences under different encoding conditions) was affected by the memory modality and the group. Effect sizes are reported in terms of partial eta squared (η_p_^2^).

## 3. Results

### 3.1. Memory Accuracy

Regarding memory accuracy, an ANOVA with memory modality (visual, audiovisual) and validity (invalid, neutral) as within-subject factors and group (ADHD, TD) as between-subject factors was conducted. The results revealed that the main effect of memory modality was significant, *F* (1, 68) = 41.85, *p* < 0.001, η_p_^2^ = 0.38, demonstrating a higher memory accuracy under the audiovisual condition (93.3%) as compared to the visual condition (87.2%). The main effect of group was also significant, *F* (1, 68) = 6.68, *p* = 0.012, η_p_^2^ = 0.09, with lower accuracy for ADHD (88.5%) than for TD children (91.9%). Additionally, only significant two-way interaction between memory modality and group was found, *F* (1, 68) = 4.35, *p* = 0.041, η_p_^2^ = 0.06. For memory modality, a post hoc analysis with Bonferroni correction revealed a significant difference between children with ADHD and TD children under the visual condition (84.5% vs. 89.8%, *p* = 0.005). No significant difference under the audiovisual condition between the two groups was observed (92.6% vs. 94.0%, *p* = 0.40). For group, a similar analysis revealed significant differences between visual and audiovisual modalities were found in children with ADHD (84.5% vs. 92.6%, *p* < 0.001), as well as in TD children (89.8% vs. 94.0%, *p* = 0.008). No other significant main effects or interaction effects were observed (*ps* > 0.17). The results suggested that the participants’ memory performance was affected by the memory modality, and the memory accuracy was much greater under the audiovisual condition than under the visual condition. In addition, the memory accuracy of TD children was greater than that of the children with ADHD under the V condition, but no difference was observed under the AV condition.

### 3.2. Search Accuracy

**Figure 2:**
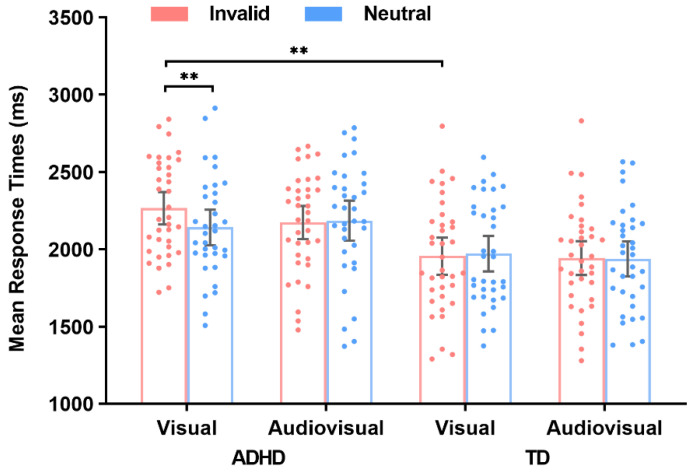
Mean response times for each condition (the mean response times are a function of memory modality, validity, and group). Error bars denote the within-subject 95% confidence intervals, as described by Morey (2008) [45]. ** *p* < 0.01.

Regarding search accuracy, search performance reached a ceiling in all conditions (above 93%). A 2 (memory modality: V vs. AV) × 2 (validity: invalid vs. neutral) × 2 (group: ADHD vs. TD) repeated-measures ANOVA on search accuracy was conducted. The results showed a significant main effect of validity, *F* (1, 68) = 4.21, *p* = 0.044, η_p_^2^ = 0.06, indicating a higher search accuracy in invalid trials (95.6%) than that in neutral trials (94.7%). The main effect of group was marginally significant, *F* (1, 68) = 3.56, *p* = 0.064, η_p_^2^ = 0.05, with lower accuracy for ADHD (94.1%) than for TD children (96.2%). However, the main effect of memory modality was not significant, *F* (1, 68) = 0.32, *p* = 0.57. Moreover, all interaction effects were not significant (*ps* > 0.39). The details of the accuracy and RTs are shown in Table 1 and Figure 2. Based on these results, the search accuracy was not affected by the memory modality, and children with ADHD committed more errors to execute visual search than TD children.

### 3.3. Search RTs

A repeated measures ANOVA was conducted on mean search RTs with the factors of memory modality, validity, and group. The results showed a significant main effect of group, *F* (1, 68) = 10.94, *p* = 0.002, η_p_^2^ = 0.14, with a slower response for ADHD (2192 ms) than for TD children (1959 ms). However, the main effect of memory modality was not significant, *F* (1, 68) = 0.92, *p* = 0.34, as was the main effect of validity, *F* (1, 68) = 2.15, *p* = 0.15. There was significant two-way interaction between validity and group, *F* (1, 68) = 4.82, *p* = 0.032, η_p_^2^ = 0.07. Importantly, the interaction between the three factors was also significant, *F* (1, 68) = 6.07, *p* = 0.016, η_p_^2^ = 0.08. Neither the interaction between memory modality and group, *F* (1, 68) = 0.01, *p* = 0.91, nor between memory modality and validity, *F* (1, 68) = 3.22, *p* = 0.077, were significant. To test whether the pattern of interaction between memory modality and validity differed in ADHD and TD children, two separate 2 (memory modality: V vs. AV) by 2 (validity: neutral vs. invalid) repeated measures ANOVA were conducted.

For ADHD children, the main effect of validity was significant, *F* (1, 34) = 5.25, *p* = 0.028, η_p_^2^ = 0.13, indicating that RTs on neutral trials (2158 ms) were faster than those on invalid trials (2226 ms). The main effect of memory modality was not significant, *F* (1, 34) = 0.48, *p* = 0.49, η_p_^2^ = 0.01. The interaction between memory modality and validity was also significant, *F* (1, 34) = 8.59, *p* = 0.006, η_p_^2^ = 0.20. A post hoc analysis with Bonferroni correction only revealed a significant difference between the invalid and neutral trials under the V condition (2281 vs. 2131 ms, *p* = 0.003), but not under the AV condition (2171 vs. 2186 ms, *p* = 0.83). For TD children, no significant main effect or interaction effects were observed (*ps* > 0.51).

We performed additional analyses to determine how the memory-driven attentional capture effect (e.g., the RTs of invalid trials minus the RTs of neutral trials differences under different encoding conditions) was affected by the memory modality and the group. A 2 (memory modality: V vs. AV) × 2 (group: children with ADHD vs. TD children) repeated-measures ANOVA was conducted. The results revealed that the main effect of group was significant, *F* (1, 68) = 4.82, *p* = 0.032, η_p_^2^ = 0.07, but not for the main effect of memory modality, *F* (1, 68) = 3.22, *p* = 0.077, indicating a greater capture effect in the ADHD group (68 ms) than in the TD group (−14 ms). The interaction between memory modality and validity was also significant, *F* (1, 68) = 6.07, *p* = 0.016, η_p_^2^ = 0.08. For memory modality, a post hoc analysis with Bonferroni correction revealed a significant difference between children with ADHD and TD children because of the V condition (150 vs. −26 ms, *p* = 0.007). No significant difference under the AV condition between the two groups was observed (−14 vs. −1 ms, *p* = 0.99). Regarding group, a similar analysis revealed a significant difference between the V and the AV condition in children with ADHD (150 vs. −14 ms, *p* = 0.018). No significant difference was found between the V and the AV condition in TD children (−26 vs. −1 ms, *p* = 0.97).

Based on these results, children with ADHD took much longer to complete the visual search than TD children, regardless of whether the validity of the search trial was invalid or neutral. In addition, for children with ADHD, the memory-driven attentional capture effect was affected by the memory modality. The capture effect of children with ADHD was significant under the V condition, but not under the AV condition. For TD children, the capture effect was not significant regardless of whether the modality of the memory sample was visual or auditory. These results suggested that memory-matched distractors capture the attention of children with ADHD under the V encoding condition possibly due to their cognitive control impairments. Semantically congruent AV encoding could compensate for the lack of cognitive control, thereby allowing children with ADHD to guide attention away from memory-matched distractors.

## 4. Discussion

The present study aimed to investigate the modulation of cognitive control over memory-driven attentional capture in children with ADHD. The study employed a dual-task paradigm in which a cue display was presented between the memory display and the search display to maximize the motivation to exert cognitive control. Analysis of search performance revealed remarkable differences when comparing the children with ADHD and TD children. Most importantly, our data indicated that the cognitive control deficit in ADHD could be improved by semantically congruent multisensory WM encoding. Specifically, for the visual encoding condition, the responses were slower in invalid trials than in neutral trials, indicating memory-driven attentional capture. Such a result was found only in children with ADHD. However, for the multimodal (AV) encoding condition, no capture effect was observed in either children with ADHD or healthy controls.

Under the unimodal (V) encoding condition, WM-related distractors captured the attention of children with ADHD but not of TD children. These contrasting effects suggested that children with ADHD had weaker cognitive control abilities than TD children and were more susceptible to the WM content matched with a distractor. This finding is consistent with previous studies. Some evidence has shown that ADHD is associated with marked difficulty in shifting and inhibition functions [46,47]. For instance, Fernandez-Ruiz et al. (2020) found that children with ADHD were worse at attentional control in prosaccade and antisaccade tasks [6]. Consistent with such behavioral results, electrophysiological studies provided further evidence that children with ADHD exhibited a smaller Pd component, a neurophysiological marker of the attentional suppression mechanism [48], in visual search tasks with distractors [29]. Furthermore, some neuroimaging evidence has indicated that children with ADHD exhibited poorer inhibition of distraction than TD children due to inefficient processing in their prefrontal cortex [49]. Together the aforementioned studies indicate a delay in the maturation of brain areas involved in cognitive control, such as frontal cortical structures [50] or impairments in cognitive control [2] in this developmental disorder. Therefore, reduced cognitive control may hinder children with ADHD from guiding attention away from memory-matched distractors.

Relatedly, it is also possible that the current results are due to energetic dysfunction in children with ADHD [51]. According to the cognitive-energetic model (CEM) suggested by Sergeant (2000, 2005), cognitive performance is not only influenced by cognitive ability but also modulated by energy factors (e.g., effort, arousal, and activation) [52,53]. For example, effort is supposedly evident in regulating the current state of the organism to meet task demands, whereas arousal is associated with signal intensity. Previous studies have found that ADHD was associated with reduced effort to meet task demands, and the regulation of arousal in accordance with task demands seems to influence attentional performance in individuals with ADHD [54,55]. Additionally, children with ADHD suffer from underarousal or underactivation, resulting in poor cognitive performance under the “normal” condition [56]. This fits our results in the way that auditory information that is semantically consistent and synchronously presented with visual WM content may raise the levels of arousal and thereby provide children with ADHD with an optimal level of arousal at which their overall performance was improved.

An interesting result of the present study was that the memory-driven attentional capture effect disappeared in children with ADHD under the condition with multisensory WM encoding, suggesting that such semantically congruent bimodal presentation could facilitate the establishment of cognitive control and then eliminate attentional capture by memory-matched distractors. The signal suppression hypothesis proposes that the actual deployment of attention depends on the relative strengths of priority signals and top-down control signals [25,26,57]. Attentional capture can be eliminated or even reversed when the top-down control signal is stronger than the priority signal. It is, therefore, concluded that the priority signal generated by multisensory WM content can be counteracted by top-down suppression in the present study. Evidence for the results that multisensory WM encoding can facilitate cognitive control over memory-driven attentional capture, in part, comes from studies on multisensory WM. Some memory studies have demonstrated that the central executive component of WM can integrate initially processed visual and auditory information from different WM subsystems into a unified multisensory representation during the encoding stage and then accelerate subsequent retrieval processing [36,58,59]. These findings can be explained by the integrated perception-cognition theory, which states that highly efficient perception processing (i.e., semantically congruent multisensory encoding) could leave more available resources for subsequent cognitive processing [60]. Considering that cognitive control over the capture of attention is implemented in part by tuning the memory representation itself [61] and that enhanced WM representations can facilitate cognitive control over memory-driven attentional capture [62], such a multisensory representation may be used to facilitate subsequent search performance. Furthermore, previous studies have indicated that there were partially shared multimodal attentional resources in the visual and auditory modalities [63,64,65]. Participants can use more attentional resources to establish sufficient cognitive control under semantically congruent multisensory encoding conditions, leading to the disappearance of attentional capture driven by memory-matched distractors.

The ability of multisensory facilitation in distractor suppression could involve brain regions involved in inhibitory control, such as the dorsolateral prefrontal cortex. Neuroimaging studies have shown that children with ADHD exhibit abnormal activation of these brain regions specifically during demanding tasks associated with inhibitory control [45,66,67]. Multisensory stimuli may compensate for the depleted recruitment of prefrontal attentional control mechanisms in children with ADHD by activating these same brain regions to recruit additional cognitive processing resources [68,69,70], thereby eliminating memory-driven attentional capture. Future research is needed to further elucidate the mechanisms by which multisensory information modulates memory-driven attention in children with ADHD.

In the present study, no advantage of WM content-related distractors was observed, regardless of whether the modality of the memory sample was visual or audiovisual in TD children, which is usually reported in studies with healthy adults [21,23]. There are two possible interpretations for this result. First, given that utilization of the WM representations that indicated the to-be-ignored features in visual search is cognitively demanding, participants may not use this additional information when there is an easier method to complete the search task [71,72]. Thus, the behavioral performance in the invalid condition was comparable with that in the neutral condition. Second, in the context of attentional control driven by ignoring distractors, it would be important to consider that some evidence indicates that memory-driven attentional capture is modulated by cognitive control [19,21,22]. Sufficient cognitive control can eliminate or even reverse attentional capture driven by memory-matched distractors. In contrast, attentional capture takes place involuntarily in the absence of fully established cognitive control. For example, a recent study found that anxiety could decrease the efficiency of cognitive control, and the higher the level of anxiety, the greater the likelihood of attentional capture by memory-matched distractors [73]. Studies examining the development of cognitive control have shown that it develops rapidly throughout childhood and is not mature until late childhood and adolescence [74]. Furthermore, a few studies have also demonstrated a slow maturation of multisensory processes for goal-irrelevant objects in school-aged children [75,76]. Therefore, it is reasonable to ascribe the absence of memory-driven attentional capture in our study to immature attentional control mechanisms in children. Considering that previewing distractors can provide strong motivation to exert cognitive control [20,44], which would encourage participants to utilize WM representations, we believe the latter interpretation to be more plausible.

Apart from poor search performance, children with ADHD performed worse than healthy controls regarding memory accuracy measures under the unimodal encoding condition. Previous studies have confirmed that WM impairment is a typical cognitive impairment in ADHD [7]. Individuals with ADHD showed abnormal functioning in brain regions associated with WM, including the bilateral dorsolateral prefrontal cortex, bilateral inferior frontal gyrus, dorsal anterior cingulate cortex, and inferior parietal lobule [8,77]. Interestingly, under the multimodal encoding condition, the memory accuracy in children with ADHD was comparable to that in TD children; that is, although multisensory encoding promoted memory retrieval in both groups, its benefits were more pronounced for the ADHD group, which was consistent with previous studies [38]. Notably, previous multisensory WM studies have found that semantically consistent multisensory encoding promotes the speed of memory retrieval rather than accuracy, as found in the present study [36,56]. We believe that this was due to the different experimental designs used. In previous multisensory studies, participants were required to perform a change detection task, in which memory retrieval accuracy was already near the top, thereby resulting in a limited number of multisensory benefits. The present study required participants to complete a visual search task while performing this task. Such a dual task made it difficult for participants to achieve high memory retrieval accuracy under the unimodal encoding condition, which, in turn, produced significant multisensory benefits. Additionally, studies using other memory tasks have also found that semantically congruent multisensory encoding can improve memory retrieval accuracy in adult and school-aged children [78,79]. Therefore, in the present study, it may not be surprising that multisensory WM encoding promoted subsequent memory performance in children with ADHD.

## 5. Strengths and Limitations

In the present study, the robust difference in the memory-driven attentional capture effect between children with ADHD and TD children provided evidence that children with ADHD were deficient in resistance to proactive interference from WM. More importantly, their performance improved overall in the semantically congruent multisensory WM encoding condition. This work may extend our understandings of the relationship between ADHD and cognitive control and provide new insight into the influence of cross-modal processing on attentional guidance. On the other hand, this may have important practical implications, especially for the intervention/training of children with ADHD. For example, multisensory stimulation can be added to attentional control or working memory training to improve the cognitive control of children with ADHD.

There are several limitations to the present study. First, the small sample size recruited for this study did not allow further analysis of the effects of ADHD subtypes and genders on cognitive control over memory-driven attentional capture. Second, we included a homogeneous sample of children with ADHD and with limited comorbidities (i.e., ODD). Therefore, it is possible that findings may be not generalizable to a more severe ADHD population. Third, due to the limitation of the experimental design, we could not analyze whether ADHD affects memory retrieval speed. Future studies need to separate the memory recognition and the visual search task.

## 6. Conclusions

In summary, the present study investigated the modulation of cognitive control over memory-driven attentional capture in children with ADHD. The results showed robust between-group differences under the unimodal (visual) WM encoding condition; that is, no memory-driven attentional capture was observed in TD children, but significant capture was found in children with ADHD. Moreover, under the semantically congruent bimodal (audiovisual) WM encoding condition, no capture was found in either children with ADHD or TD children. The present study provided evidence that children with ADHD were deficient in resistance to proactive interference from WM, and these deficits could be improved by semantically congruent multisensory WM encoding.

## Figures and Tables

**Table 1:** Mean of average search RTs on correct trials and mean accuracy of search task and memory task (with standard errors in parentheses) for each condition.

	Memory Modality	Validity	Search RT (ms)	Search Accuracy (%)	Memory Accuracy (%)
ADHD	Visual	Invalid	2281 (51)	94.4 (1.2)	84.2 (1.6)
		Neutral	2131 (56)	93.6 (1.2)	84.8 (1.8)
	Audiovisual	Invalid	2171 (55)	94.5 (1.0)	92.8 (1.2)
		Neutral	2186 (62)	93.8 (1.3)	92.3 (1.2)
TD	Visual	Invalid	1957 (61)	97.2 (0.7)	89.9 (1.1)
		Neutral	1983 (56)	96.1 (0.9)	89.7 (1.3)
	Audiovisual	Invalid	1948 (56)	96.3 (0.8)	95.0 (0.7)
		Neutral	1948 (54)	95.3 (1.0)	92.9 (0.9)
		Neutral	2015 (60)	95.3 (1.0)	92.6 (0.9)

## Data Availability

The data is available from the corresponding author upon reasonable request.
